# The Effects of Initial Procalcitonin Levels on Mortality Rates in Geriatric Patients Undergoing Surgery

**DOI:** 10.7759/cureus.7613

**Published:** 2020-04-10

**Authors:** Belkiz Ongen İpek, Aslı Karadeniz, Mustafa Erinc Sitar

**Affiliations:** 1 Medical Biochemistry, Maltepe University Faculty of Medicine, İstanbul, TUR; 2 İnfectious Disease, Maltepe University, Istanbul, TUR; 3 Medical Biochemistry, Maltepe University Faculty of Medicine, Istanbul, TUR

**Keywords:** geriatric medicine, mortality, procalcitonin, surgery

## Abstract

Introduction

The aim of the current study is to investigate the relationship between mortality rate in geriatric patients undergoing surgery with preoperative serum levels of procalcitonin, C-reactive protein, and erythrocyte sedimentation rate.

Methods

This was a single-center retrospective study, including three groups with 101 patients, who are older than 65 years of age. A retrospective investigation was carried out from the laboratory information system for all groups from January to December 2018. Group 1 included patients who had surgery and then mortality within 30 days after surgery. Group 2 included hospitalized patients who had surgery and no mortality within 30 days after surgery. Group 3 included outpatient patients, who had suspicion for a bacterial infection and then no surgery or no mortality within 30 days.

Results

When three group comparisons were made for procalcitonin, C-reactive protein, and erythrocyte sedimentation rate values, the p-value of one-way analysis of variance (ANOVA) was higher than 0.05 for procalcitonin and lower than 0.05 for C-reactive protein and erythrocyte sedimentation rate, suggesting that one or more groups were significantly different. When post-hoc multiple comparison methods were applied, there were statistically significant differences between Groups 1 and 3 for C-reactive protein and erythrocyte sedimentation rate.

Conclusions

Procalcitonin levels do not predict mortality following surgery. C-reactive protein and erythrocyte sedimentation rate are more useful biomarkers predicting mortality in geriatric patients undergoing surgery.

## Introduction

According to a World Health Organization (WHO) report, aging is defined as a process that progresses with the accumulation of gradual damage at the molecular and cellular levels and continues with a reduction in physiological and cognitive capacity, eventually resulting in inevitable death [1]. Although life span has not changed in the present century, life expectancy is increasing day by day and the average life expectancy in today's average world society is over 60 years old [2]. High success rates in vaccination, increased access to antibiotic use, rapid access to the health system, effective communication and logistic systems, and increased success rates in pharmacological treatments of chronic diseases are positively reflected in these statistics. As the age of the population increases gradually, the prevalence of diseases such as Alzheimer's, Parkinson's, malignancies, Type 2 diabetes mellitus, cataracts, osteoporosis, and hypertension is increasing. Infections should be included in this list as well. The elderly population has higher numbers of ongoing challenges due to atypical and/or ambiguous clinical presentations as compared to younger peers. In addition, they have increased comorbidity and death risk together with low immunological status [3]. It is crucial to identify geriatric patients who have an increased risk of mortality and develop strategies to prevent the outcome. Severity status grading systems, which are routinely applied for critically ill people like Acute Physiologic Assessment and Chronic Health Evaluation (APACHE) II, Mortality Prediction Model (MPM), Sequential Organ Failure Assessment (SOFA), and Simpliﬁed Acute Physiology Score (SAPS II) may have irrelevant scores in elderly and/or septic patients. If patients at risk for sepsis and mortality are detected more quickly, there may be a chance to take precautions and reduce mortality rates. In this fundamental problem, medical laboratories can contribute to clinical practice with biomarkers that mirror general health status.

Procalcitonin (ProCT) entered the scientific literature in the 1970s [4]. It was found out to be a higher molecular weight precursor of calcitonin. In the early 90s, it has been found out that it has a linear relation with microbial invasion and gives information for the differentiation of viral infections from bacterial ones [5]. In current practice, ProCT gives valuable data in the primary diagnosis of bacterial sepsis and guide antibiotic therapy [6-8]. It also assists the clinician in the selection of pharmacological agents, monitoring the effective response to treatment and duration of administration. For critical reasons such as these, the use of ProCT is increasing day by day all over the world. However, more detailed assessments of the performance of the ProCT test in geriatric patients are required. Actually, when the clinician suspects a severe bacterial infection, ProCT levels can enlighten the course. However, the situation may not be so simple in elderly patients [9]. In the literature, there are publications with different results for the relationship between ProCT and mortality. Higashikawa et al. investigated ProCT levels in the geriatric population with suspected bacterial infection [10]. They found that 30-day mortality was importantly elevated with PCT levels >0.5 ng/mL. On the other hand, Szakmary and Molnar revealed different data in their study [11]. They stated that ProCT levels do not yield enough clinical information relevant to mortality rates after major operations. Similar findings also exist for other important markers such as erythrocyte sedimentation rate (ESR) and C-reactive protein (CRP). It is well-known that CRP is a serum biomarker of an acute-phase reaction and can be elevated in inflammation, bacterial infection, and trauma [12-13]. But there is a lack of strong evidence about the prognostic value of serum biomarkers after surgery in elderly people. The aim of the current study is to investigate the relationship between mortality rate in geriatric patients undergoing surgery with the preoperative serum levels of ProCT, CRP, and ESR.

## Materials and methods

**Figure 1:**
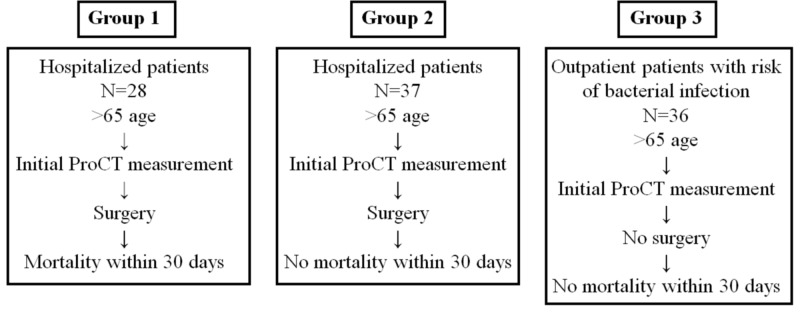
Flow chart of patients recruited in the present study (ProCT) ProCT: procalcitonin

This study was approved by the local clinical ethics board of Maltepe University Faculty of Medicine, Istanbul (issue number 2019/900/15). We conducted a retrospective study including three groups - 101 patients, who are older than 65 years of age, admitted to Maltepe University Educational and Research Hospital, Istanbul. The retrospective investigation was accomplished from the laboratory information system for all groups from January to December 2018. Group 1 included patients who had surgery and then mortality within 30 days after surgery (n=28). Group 2 included hospitalized patients who had surgery and no mortality within 30 days after surgery. Group 3 included outpatient patients who had suspicion of bacterial infection and then no surgery or no mortality within 30 days (Figure 1). Phlebotomies were applied on the first day of admission between 8 am to 10 am after overnight fasting according to the standard venipuncture quality procedures of the hospital. All samples were centrifuged after venous blood sampling. Serum ProCT levels were measured by Roche Hitachi E170 (Basel, Switzerland) using the immunoassay method after centrifugation. Blood urea nitrogen (BUN), creatinine, albumin, total protein, aspartate aminotransferase (AST), and alanine aminotransferase (ALT) activity levels were measured by Siemens (Munich, Germany) Dimension RXLmax after centrifugation according to the instructions of the manufacturer. The sedimentation rate was analyzed by Electalab (Forli Italy) Sedy40 and complete blood count was measured by Sysmex (Kobe Japon) XT2000i.

Biostatistical analyses were performed using SPSS version 21 (SPSS Inc., Chicago, Illinois) and p-value <0.05 was considered statistically significant. The comparison between the groups in the parametric results was made by analysis of variance (ANOVA. When there was a difference between the groups, post-hoc multiple comparison methods Tukey, Bonferroni, and Scheffé tests were used to determine which group caused the difference.

## Results

**Figure 2:**
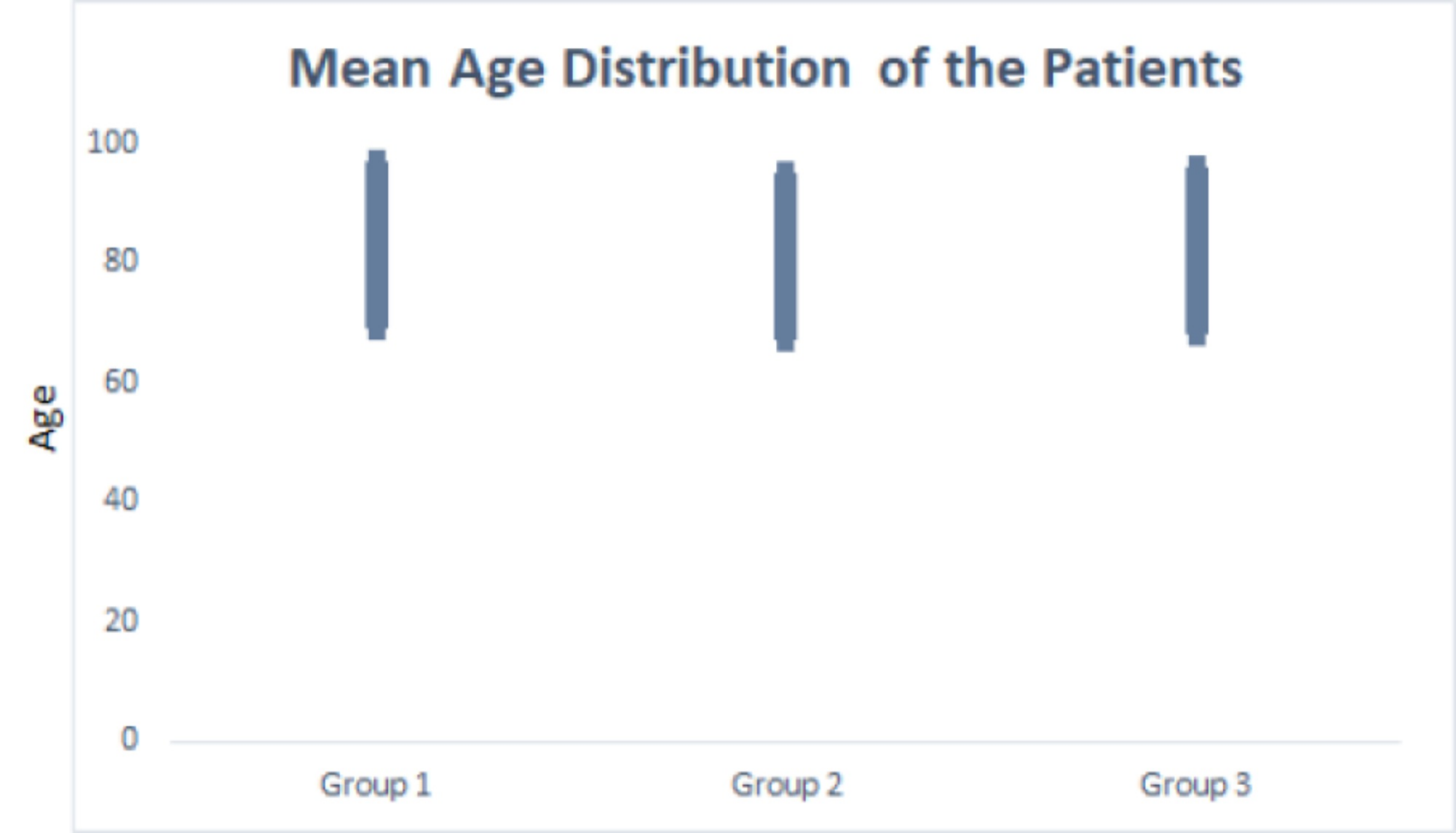
Mean ages of the groups and standard deviations

A total of 101 geriatric patients were included in the study; the mean age of patients was demonstrated in Figure 2 (higher than 80 years for the entire study group). ProCT mean±SD values were found to be 7.2±21 for Group 1, 3.93±13 for Group 2, and 0.58±1.2 for Group 3. CRP mean±SD values were obtained as 9.4±9 for Group 1, 7.3±7 for Group 2, and 5.5±5 for Group 3. ESR mean±SD values were obtained as 63±22 for Group 1, 51±26 for Group 2, and 44±22 for Group 3 (Table 1). The groups did not differ in ProCT levels [F(2, 113)=2.37, p=0,0983]. Significant differences were found for CRP [F(2, 104)=3.30, p=0,0409] and ESR levels [F(2, 104)=5.58, p=0.0050], indicating significant difference among groups. Tukey’s post-hoc test demonstrated significantly lower CRP and ESR levels in Group 3 in comparison to Group 1 (p<0.05 and p<0.001, respectively) (Table 2). Biochemical and immunoassay parameters of all groups were shown in Table 1 together with neutrophil, lymphocyte, thrombocyte counts, blood urea nitrogen (BUN), creatinine, AST, ALT, albumin, ProCT, CRP, and ESR.

**Table 1:** Biochemical parameters of all groups together with neutrophil, lymphocyte, thrombocyte counts, BUN, creatinine, AST, ALT, albumin, ProCT, ESR, and CRP BUN: blood urea nitrogen; AST: aspartate aminotransferase; ALT: alanine aminotransferase; ProCT: procalcitonin; ESR: erythrocyte sedimentation rate; CRP: C-reactive protein

	Group 1 (Mean±SD)	Group 2 (Mean±SD)	Group 3 (Mean±SD)
BUN (mg/dL)	52,17±41	28±17	40,73±34
Creatinine (mg/dL)	1,74±1,3	1,01±0,6	1,65±1
AST (U/L)	63,3±82	26,97±18,7	80,46±284,6
ALT (U/L)	51,5±68	23,8±11	69,21±181,5
Albumin (mg/dL)	2,37±0,6	2,61±0,5	2,9±0,6
Neutrophil (10^3^/µL)	9,08±6	8,6±4,8	6,8±4
Lymphocyte (10^3^/µL)	1,3±8	1,5±7	1,4±6
Thrombocyte (10^3^/µL)	212,3±112	212,2±94	243,2±107
ProCT (µg/L)	7,2±21	3,93±13	0,58±1,2
ESR	63±22	51±26	44±22
CRP (mg/L)	9,4±9	7,3±7	5,5±5

**Table 2:** CRP, ProCT, and sedimentation tests, one-way ANOVA, and post-hoc comparison results of patients and group comparisons CRP: C-reactive protein; ProCT: procalcitonin; ANOVA: analysis of variance

CRP			ProCT			Sedimentation		
One-way ANOVA *p-value: 0,0409	Post-hoc Pair Comparisons		One-way ANOVA *p-value: 0,0983	Post-hoc Pair Comparisons		One-way ANOVA p-value: 0,005	Post-hoc Pair Comparisons	
	Group I and II	insignificant		Group I and II	insignificant		Group I and II	insignificant
	Group I and III	* p<0.05		Group I and III	insignificant		Group I and III	** p<0.001
	Group II and III	insignificant		Group II and III	insignificant		Group II and III	insignificant

## Discussion

Our study indicated that ProCT levels did not predict mortality following surgery, however, CRP and ESR were more useful biomarkers predicting mortality in geriatric patients undergoing surgery.

There are distinctive studies about ProCT in the elderly population and mortality predictions in the current medical literature. Yu H et al. searched the levels of ProCT and the 30-day mortality rate, together with a quick sequential organ failure assessment (SOFA) score, in hospitalized patients. They found a significant correlation between those parameters unlike our findings [14]. Fritz et al. assessed ProCT levels and mortality after cardiopulmonary bypass surgery [15]. They concluded that elevated levels of ProCT (>2.5 ngl-1) are predictive of 28-day mortality. On the contrary, Szakmany stated that ProCT levels do not predict mortality following major abdominal surgery [11]. This study contains findings consistent with our study. Clementi et al. studied presepsin and ProCT levels for mortality in cardiac surgery patients [16]. They found that the predictive value for in-hospital, 30-day, and six-month mortality was higher for presepsin. Kim et al. found that ProCT levels were associated with pneumonia intensity but not with mortality in elderly patients with community-acquired pneumonia [17]. Vallet H et al showed a significant association of high ProCT values with mortality within 30 days for geriatric patients after orthopedic surgery [18]. Stucker et al. investigated ProCT in detecting infection in elderly patients [19]. They concluded that increased ProCT levels >0.5 ng/mL is associated with infection in the geriatric population. Lee et al. studied ProCT levels with bacterial infections in the geriatric population [20]. They concluded that ProCT can be used to exclude sepsis in the geriatric population. In our study, we found that ProCT levels were not giving information about one-month mortality properly.

Increased serum CRP levels are independently determinative for elevated mortality and poor clinical outcome in elderly patients with vascular diseases such as atherosclerosis, coronary heart disease and stroke [21-23]. Kim et al. investigated preoperative CRP levels as a one-year mortality predictor in elderly patients who underwent hip fracture surgery [24]. They found that one-year mortality was significantly increased in patients whose CRP levels were higher than 10 mg/dL. All these results correlate very well with the current study. On the other hand, Beloosesky et al. found no association between CRP levels and six-month mortality in geriatric patients who underwent hip-fracture surgery [25]. These controversial findings raise a question about the predictive value of CRP.

ESR is the measurement of red blood cell aggregation, which rises when an augmentation in the total mass exceeds the increase in volume [26]. ESR is a biomarker of inflammation and can be elevated in rheumatoid arthritis, Crohn’s disease, ulcerative colitis, and cancer [27]. Shibuya et al. searched the relationship between ESR and the postoperative mortality of patients with colorectal cancer [27]. They found that preoperative high levels of ESR (>40 mm/h) can predict poorer postoperative survival in patients with colorectal cancer. Seong et al. investigated the relationship between inflammatory biomarkers such as CRP, ESR, white blood cell count (WBC), neutrophil/ lymphocyte ratio and postoperative survival of patients with colorectal cancer [26]. They concluded that CRP, ESR, and the neutrophil/lymphocyte ratio were significantly associated with survival relevant to the current study.

The main limitations of our study are that it is a single-center study, with a variety of operations, and ProCT relevance to bacterial infections rather than viral ones. However, if the mean age of patients is considered, this study is highly valuable to guide future studies.

## Conclusions

It is necessary to focus on “healthy aging” rather than preventing or stopping aging. Because clinical laboratories contribute more than 70% to clinical processes, they must assume their role in healthy aging as well. Prediction of bad outcomes can be very beneficial for both health care professionals and the elderly themselves. We suggest day-by-day measurement of ProCT values for every geriatric patient and manufacturing a systematic panel using other grading systems. And it should always be kept in mind that every geriatric population is special clinically.
